# Author Correction: ALKBH5-mediated m6A modification of IL-11 drives macrophage-to-myofibroblast transition and pathological cardiac fibrosis in mice

**DOI:** 10.1038/s41467-024-49969-5

**Published:** 2024-07-03

**Authors:** Tao Zhuang, Mei-Hua Chen, Ruo-Xi Wu, Jing Wang, Xi-De Hu, Ting Meng, Ai-Hua Wu, Yan Li, Yong-Feng Yang, Yu Lei, Dong-Hua Hu, Yan-Xiu Li, Li Zhang, Ai-Jun Sun, Wei Lu, Guan-Nan Zhang, Jun-Li Zuo, Cheng-Chao Ruan

**Affiliations:** 1 Department of Physiology and Pathophysiology, Shanghai Key Laboratory of Bioactive Small Molecules, State Key Laboratory of Medical Neurobiology, School of Basic Medical Sciences, and Jinshan Hospital, Fudan University, Shanghai, China; 2 Institute of Metabolism and Regenerative Medicine, Shanghai Sixth People’s Hospital Affiliated to Shanghai Jiao Tong University School of Medicine, Shanghai, China; 3 Minhang Hospital and School of Pharmacy, Key Laboratory of Smart Drug Delivery Ministry of Education, State Key Laboratory of Molecular Engineering of Polymers, Fudan University, Shanghai, China; 4 Department of Cardiology, RuiJin Hospital/LuWan Branch, Shanghai Jiao Tong University School of Medicine, Shanghai, China; 5 Department of Critical Care Medicine, The First Affiliated Hospital of Nanjing Medical University, Nanjing, China; 6 Department of Cardiology and Institute for Developmental and Regenerative Cardiovascular Medicine, Xinhua Hospital, Shanghai Jiaotong University School of Medicine, Shanghai, China; 7 Department of Cardiology, Zhongshan Hospital, Fudan University, Shanghai Institute of Cardiovascular Diseases, Shanghai, China; 8 Department of Immunology, Nanjing Medical University, Nanjing, Jiangsu China; 9 Department of Geriatrics, Ruijin Hospital, Shanghai Jiao Tong University School of Medicine, Shanghai, China

**Keywords:** Monocytes and macrophages, Epigenetics, Systems analysis, Heart failure

Correction to: *Nature Communications* 10.1038/s41467-024-46357-x, published online 5 March 2024

The original version of this Article contained an error in Author affiliation, in which the author ‘Wei Lu’ should have been associated with affiliation ‘3’, but was incorrectly associated with affiliation ‘2’. This has been corrected in the both the HTML and the PDF versions.

