# Supplementary material for: ALKBH5-mediated m6A modification of IL-11 drives macrophage-to-myofibroblast transition and pathological cardiac fibrosis in mice
Source: Nat Commun. 2024 Mar 5;15:1995. doi: 10.1038/s41467-024-46357-x (PMC10914760; doi:10.1038/s41467-024-46357-x)
Supplement: Supplementary file 1 — Supplementary Information [file 41467_2024_46357_MOESM1_ESM.pdf]

Supplementary information

ALKBH5-mediated m6A modification of IL-11 drives macrophage-to-myofibroblast transition and pathological cardiac fibrosis in mice

Tao Zhuang[1†], Mei-Hua Chen[1,2†], Ruo-Xi Wu[1], Jing Wang[1], Xi-De Hu[1], Ting Meng[1], Ai-Hua Wu[3], Yan Li[4], Yong-Feng Yang[1], Yu Lei[1], Dong-Hua Hu[1], Yan-Xiu Li[5], Li Zhang[6], Ai-Jun Sun[7], Wei Lu[2*], Guan-Nan Zhang[8*], Jun-Li Zuo[9*], Cheng-Chao Ruan[1*]

1 Department of Physiology and Pathophysiology, Shanghai Key Laboratory of Bioactive Small Molecules, State Key Laboratory of Medical Neurobiology, School of Basic Medical Sciences, and Jinshan Hospital, Fudan University, Shanghai, China.
2 Institute of Metabolism and Regenerative Medicine, Shanghai Sixth People's Hospital Affiliated to Shanghai Jiao Tong University School of Medicine, Shanghai, China.
3 Minhang Hospital and School of Pharmacy, Key Laboratory of Smart Drug Delivery Ministry of Education, State Key Laboratory of Molecular Engineering of Polymers, Fudan University.
4 Department of Cardiology, RuiJin Hospital/LuWan Branch, Shanghai Jiao Tong University School of Medicine, Shanghai, China.
5 Department of Critical Care Medicine, The First Affiliated Hospital of Nanjing Medical University, Nanjing, China.
6 Department of Cardiology and Institute for Developmental and Regenerative Cardiovascular Medicine, Xinhua Hospital, Shanghai Jiaotong University School of Medicine, Shanghai, China
7 Department of Cardiology, Zhongshan Hospital, Fudan University, Shanghai Institute of Cardiovascular Diseases, China
8 Department of Immunology, Nanjing Medical University, Nanjing, Jiangsu, China
9 Department of Geriatrics, Ruijin Hospital, Shanghai Jiao Tong University School of Medicine, Shanghai, China.

One-sentence summaries: Macrophage ALKBH5 regulates pathological cardiac fibrosis

†Equally contributing authors: Tao Zhuang, Mei-Hua Chen

* Corresponding author:
Email: ruancc@fudan.edu.cn (C.-C.R.);
zuo-junli@163.com (J.-L.Z.);
gnzhang@njmu.edu.cn (G.-N.Z.);
wlu@fudan.edu.cn (W.L)

Supplemental Figures

Supplementary Figure 1

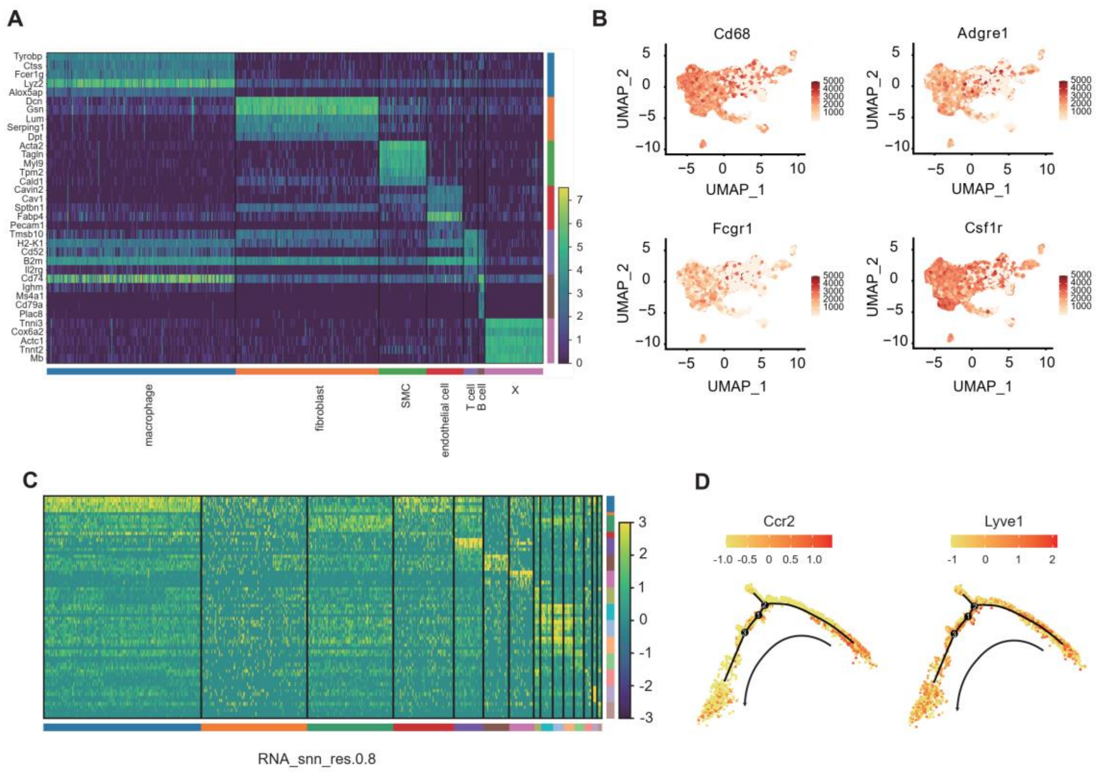

Supplementary Figure 1. scRNA sequencing reveals macrophage-myofibroblast transition induced by Ang II. A, Heatmap of the top most differentially expressed genes in each cluster from Fig. 1B**. B**, Feature plots depicting single-cell gene expression of macrophage genes visualized on the UMAP dimensionality reduction plots. **C**, Heatmap of the top most differentially expressed genes in each cluster from Fig. 1C**. D,** The Ccr2 and Lyve1 expression in pseudotemporal trajectory (Monocle) analysis from cluster macrophages to myofibroblasts.

Supplementary Figure 2

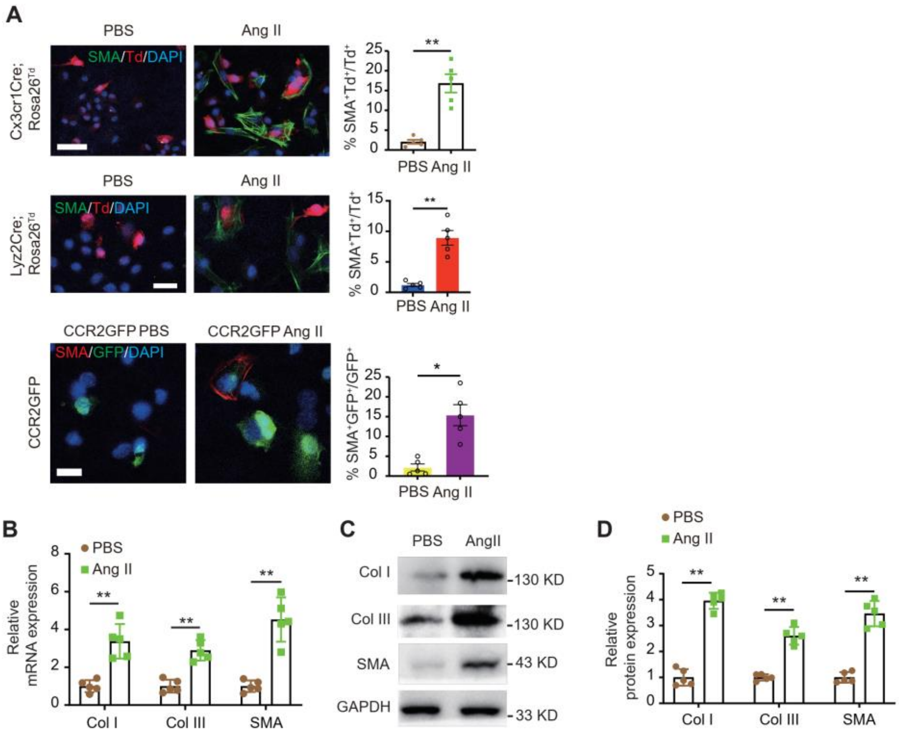

Supplementary Figure 2. Ang II increases macrophage-myofibroblast transition *in vitro* cultured macrophages. A, Representative immunofluorescent images (left) and quantification (right) of SMA^+ cells in cultured Td^+ macrophages cells from *Cx3cr1Cre; Rosa26^{Td}* and *Lyz2Cre;Rosa26^{Td}* mice, or GFP+ macrophages cells from *CCR2GFP* mice, with and without Ang II infusion. n=5. Scale bar, 100 µm. **B,** Expression of Col I, Col III and SMA by qPCR in cardiac Td+ cells from *Cx3cr1Cre; Rosa26^{Td}* mice with and without Ang II infusion. n=5. **C**, Representative images of western blot showing Col I, Col III and SMA expression in cardiac Td+ cells from *Cx3cr1Cre; Rosa26^{Td}* mice with and without Ang II infusion. n=5. **D**, Quantification of Col I, Col III and SMA expression in cardiac Td+ cells from *Cx3cr1Cre; Rosa26^{Td}* mice with and without Ang II infusion. n=5. All data are presented as mean ± standard error mean. Data in (A), (B) and (D) were analyzed by two-tailed unpaired Student's t test. *P<0.05, **P<0.01.

Supplementary Figure 3

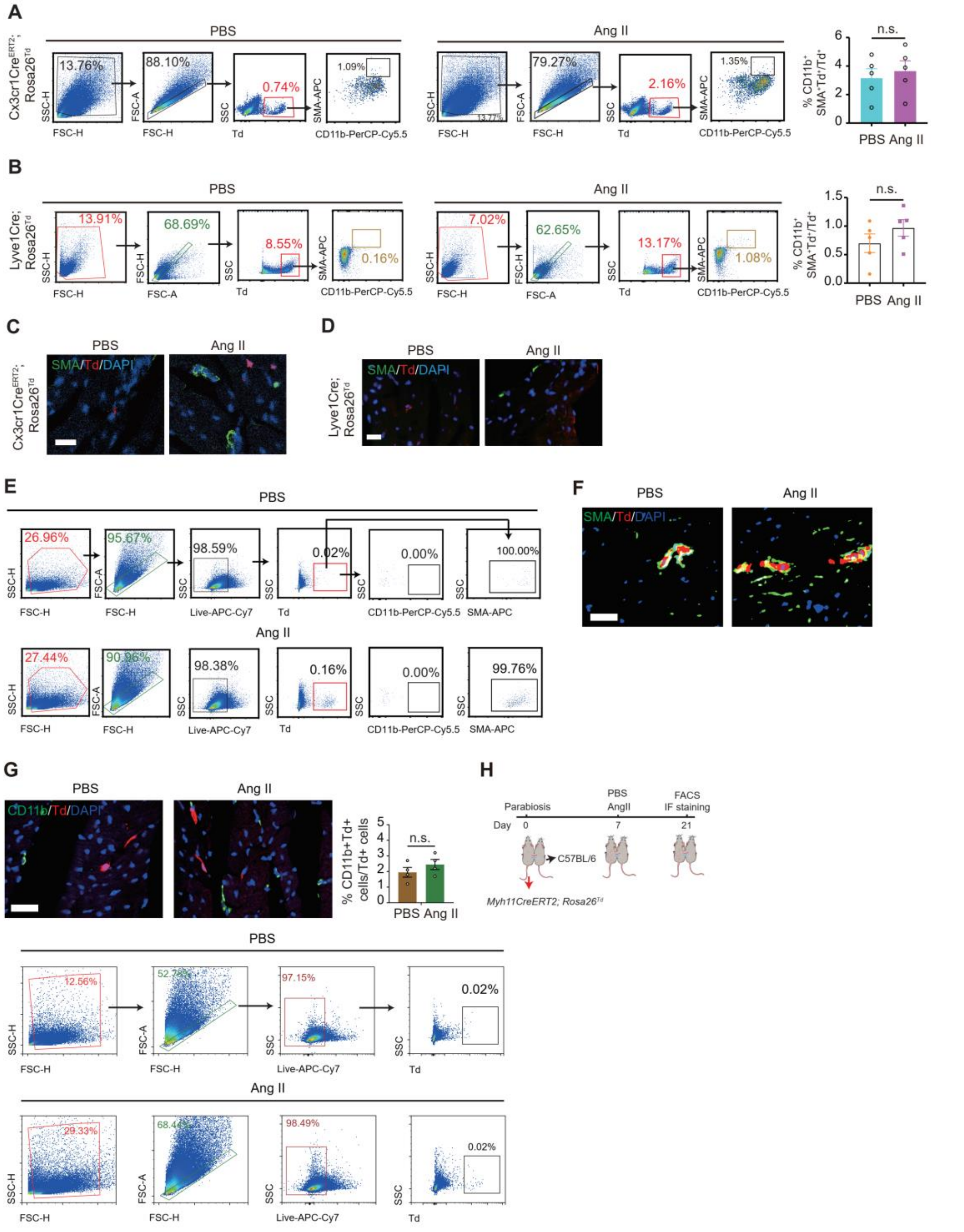

Supplementary Figure 3. Cardiac resident macrophages do not transit into myofibroblasts under hypertension showed in the *Cx3cr1Cre^{ERT2}* and Lyve1Cre lineage tracing mice. A, Representative flow cytometry analyses of cardiac CD11b$^+$SMA$^+$ cells gated on Td$^+$ cells from PBS (left) and Ang II (middle) treated *Cx3cr1Cre$^{ERT2;}$ Rosa26^{Td}* mice with quantification at right. n=5. **B,** Representative flow cytometry analyses of cardiac CD11b$^+$SMA$^+$ cells gated on Td$^+$ cells from PBS (left) and Ang II (middle) treated *Lyve1Cre;Rosa26^{Td}* mice with quantification at right. n=5. **C**, Representative immunofluorescent images of SMA$^+$Td$^+$ cells of cardiac

tissues from *Cx3cr1Cre*^{ERT2}*; Rosa26*^{Td} mice with and without Ang II infusion, with quantification at right. Scale bar, 100 µm. **D**, Representative immunofluorescent images of $SMA^{+}Td^{+}$ cells of cardiac tissues from *Lyve1Cre;Rosa26*^{Td} mice with and without Ang II infusion, with quantification at right. Scale bar, 100 µm. **E,** Representative images of flow cytometry analyses of CD11b+ and SMA+ cells gated on Td+ cells in hearts *Myh11Cre*^{ERT2}*; Rosa26*^{Td} mice with control PBS or Ang II treatment. **F**, Representative immunofluorescent images of SMA in hearts from PBS and Ang II treated *Myh11Cre*^{ERT2}*; Rosa26*^{Td} mice (n=4), with quantification at right. Scale bar, 100 µm. **G**, Representative immunofluorescent images of CD11b in hearts from PBS and Ang II treated *Myh11Cre*^{ERT2}*; Rosa26*^{Td} mice (n=4), with quantification at right. Scale bar, 100 µm. **H**, Representative images of flow cytometry analyses of Td+ cells gated on live cells in hearts from C57BL/6 mice conjoined with *Myh11Cre*^{ERT2}*; Rosa26*^{Td} mice with control PBS or Ang II. All data are presented as mean ± standard error mean. Data in (A), (B) and (G) were analyzed by two-tailed unpaired Student's t test. n.s. indicates nonsignificant. *P<0.05, **P<0.01.

Supplementary Figure 4

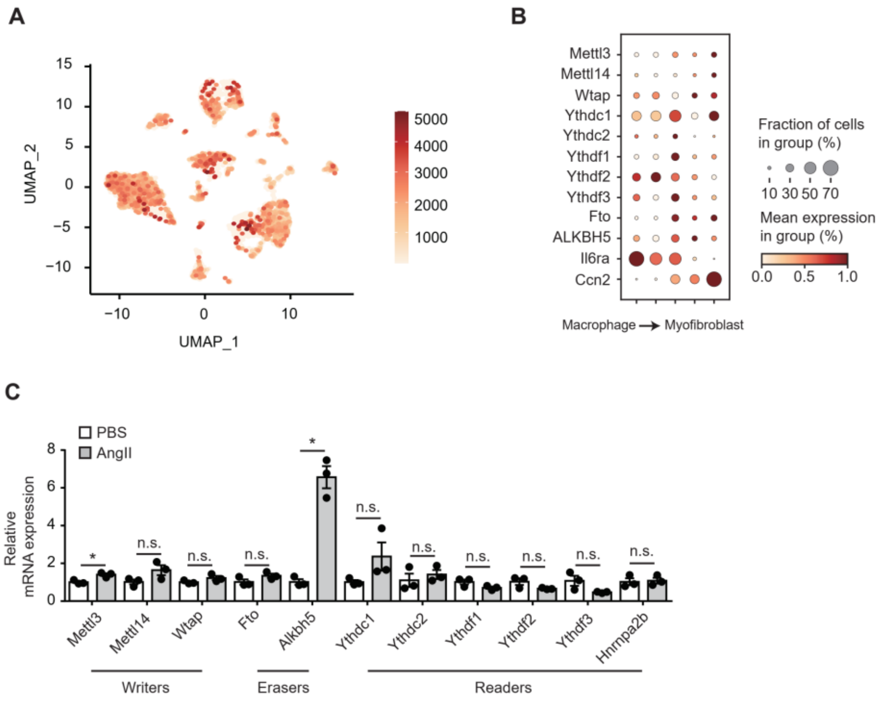

Supplementary Figure 4. ALKBH5 upregulation is involved in hypertension-induced cardiac MMT. A, Dot plots depicting single-cell gene expression of ALKBH5 in the total cardiac cell clusters visualized on the UMAP dimensionality reduction analysis. **B,** Dot plot of m6A genes for each cluster. **C**, qPCR analysis of mRNA expression levels of m6A genes in cardiac Td+ cells from *Cx3cr1Cre; Rosa26*^{Td} mice. Error bars indicate mean ± SEM. n=3. All data are presented as mean ± standard error mean. Data in (C) were analyzed by two-tailed unpaired Student's t test. n.s. indicates nonsignificant. *P<0.05.

Supplementary Figure 5

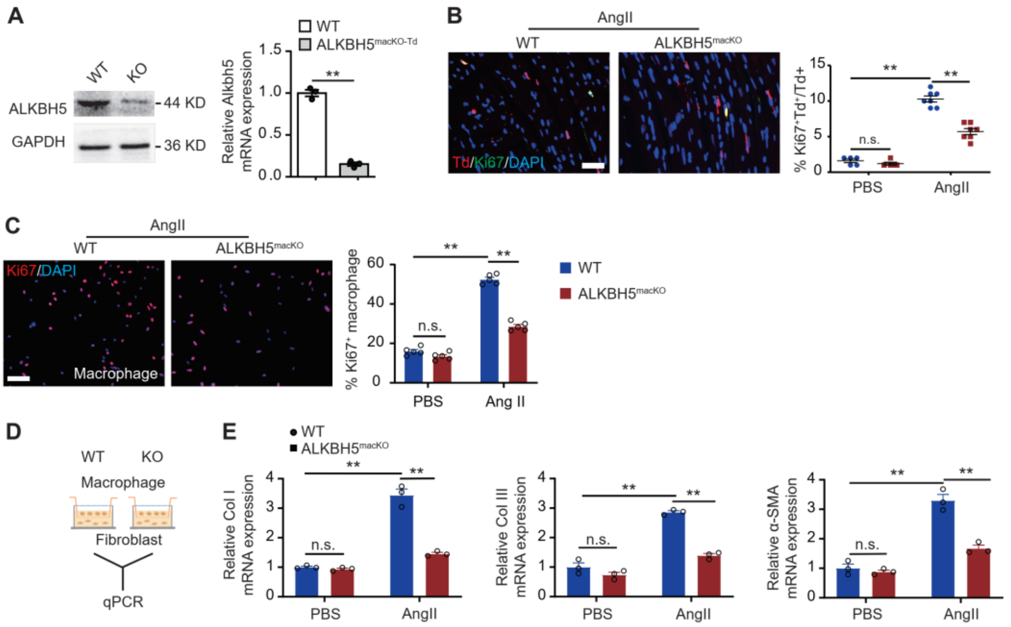

Supplementary Figure 5. ALKBH5 deletion in macrophages inhibits MMT and myofibroblast proliferation with Ang II stimulation. A, Expression of ALKBH5 in cardiac Td^{+} cells from *WT* and *ALKBH5^{macKO}* mice assessed by western blot (left) and qPCR (right). n=3. **B**, Representative immunofluorescent images and quantification of Ki67^{+} cells in Td^{+} cells of cardiac tissues from *WT* and *ALKBH5^{macKO}* mice. n =5-7. Scale bar, 100 μm. **C**, Representative images and quantification of Ki67 positive cells in *WT* and *ALKBH5^{macKO}* macrophages with and without Ang II treatment. n=5. Scale bar, 100 μm. **D**, Schematic diagram of an *in vitro* experiment to show that the effect of macrophage ALKBH5 deletion on the cultured fibroblasts. **E,** qPCR analysis of ECM genes collagen I and III and SMA in myofibroblasts treated with condition medium of macrophages. n=3. All data are presented as mean ± standard error mean. Data in (A) were analyzed by two-tailed unpaired Student's t test. n.s. indicates nonsignificant. Data in (B) were analyzed by two-way ANOVA followed by Tukey post-hoc tests. Data in (C) and (E) were analyzed by one-way ANOVA followed by Tukey post-hoc tests. n.s. indicates nonsignificant. **P<0.01.

Supplementary Figure 6

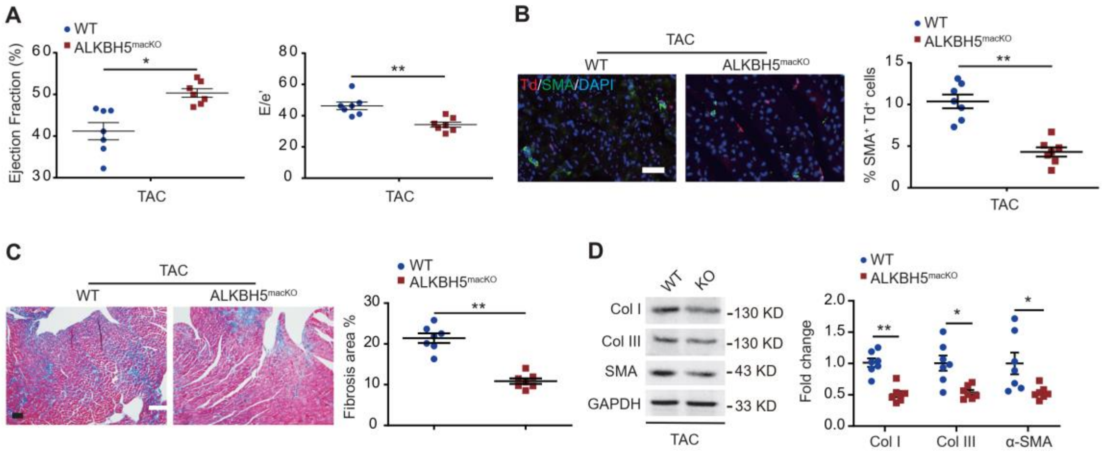

Supplementary Figure 6. ALKBH5 deletion in Cx3cr1 derived lineage improves cardiac dysfunction under pressure overload-induced cardiac remodeling. A, Quantification analysis of ejection fraction and E/e' in TAC induced *WT* and *ALKBH5*$^{macKO\text{-}Td}$ mice by echocardiography. n =7. **B,** Representative immunofluorescent images and quantification of SMA+ cells in cardiac Td+ cells from TAC induced *WT* and *ALKBH5*$^{macKO\text{-}Td}$ mice. n =7. Scale bar, 100 μm. **P<0.01. **C,** Representative images and quantification of Masson trichrome staining in cardiac tissue from TAC induced mice. n =7. Scale bar, 100 μm. **D,** Representative images and quantification of the collagen (Col) types I and III and α-SMA expression by western blot. n=7. All data are presented as mean ± standard error mean. Data in (A), (B), (C) and (D) were analyzed by two-tailed unpaired Student's t test. n.s. indicates nonsignificant. *P<0.05, **P<0.01.

Supplementary Figure 7

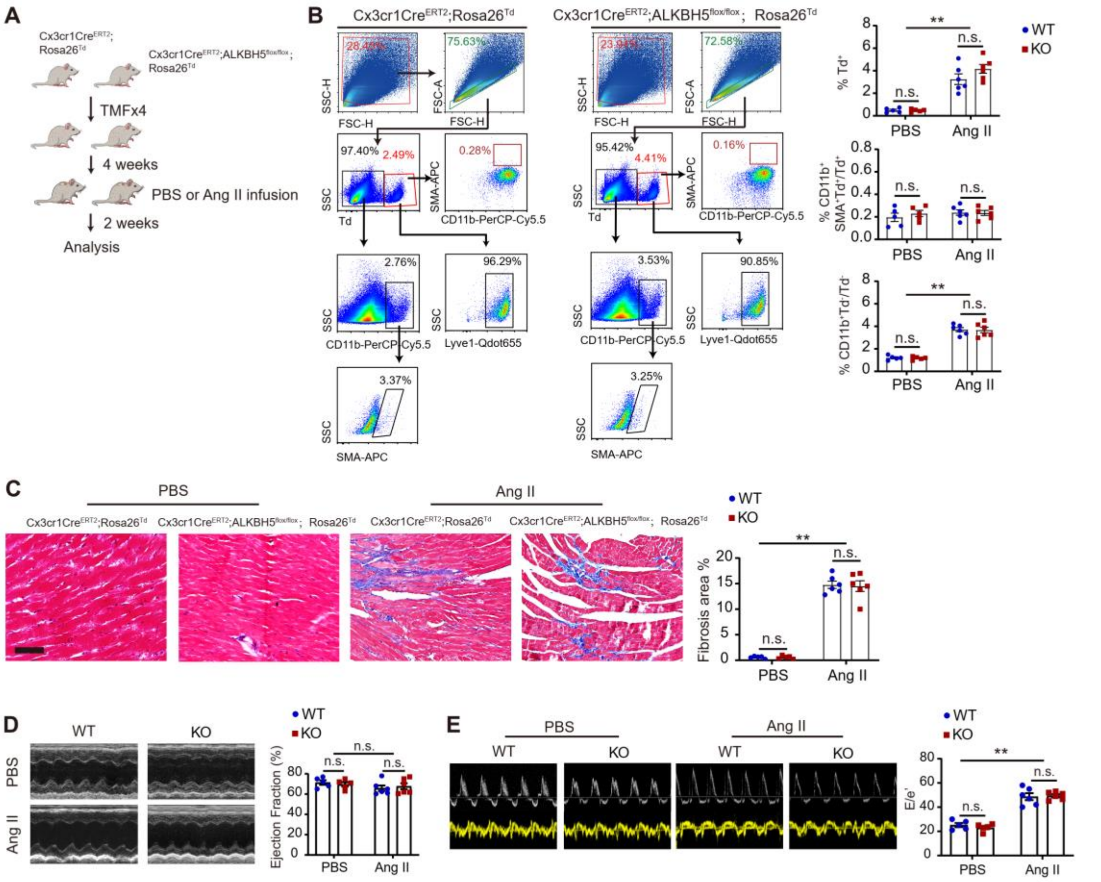

Supplementary Figure 7. ALKBH5 deletion in cardiac resident macrophages has no effects on MMT and cardiac fibrosis and dysfunction. A, Diagram of deletion ALKBH5 in cardiac resident macrophages. **B**, Representative flow cytometry analyses of cardiac CD11b$^+$SMA$^+$ cells gated on Td$^+$ cells from PBS and Angiotensin II treated *Cx3cr1Cre^{ERT2};Rosa26^{Td}* and *Cx3cr1Cre^{ERT2};ALKBH5$^{flox/flox}$;Rosa26^{Td}* mice, with representative images at left and quantification at right. n=5. **C**, Representative images of Masson trichrome staining in cardiac tissue from PBS and Angiotensin II treated *Cx3cr1Cre^{ERT2};Rosa26^{Td}* and *Cx3cr1Cre^{ERT2};ALKBH5$^{flox/flox}$;Rosa26^{Td}* mice, with quantification of positive fibrotic area at right. Scale bar, 100 μm. **D**, Representative echocardiography images and quantification of ejection fraction of above mice. **E**, Representative echocardiography images and quantification of E/e' of above mice. All data are presented as mean ± standard error mean. Data in (B), (C), (D) and (E) were analyzed by two-way ANOVA followed by Tukey post-hoc tests. n.s. indicates nonsignificant. **P<0.01.

Supplementary Figure 8

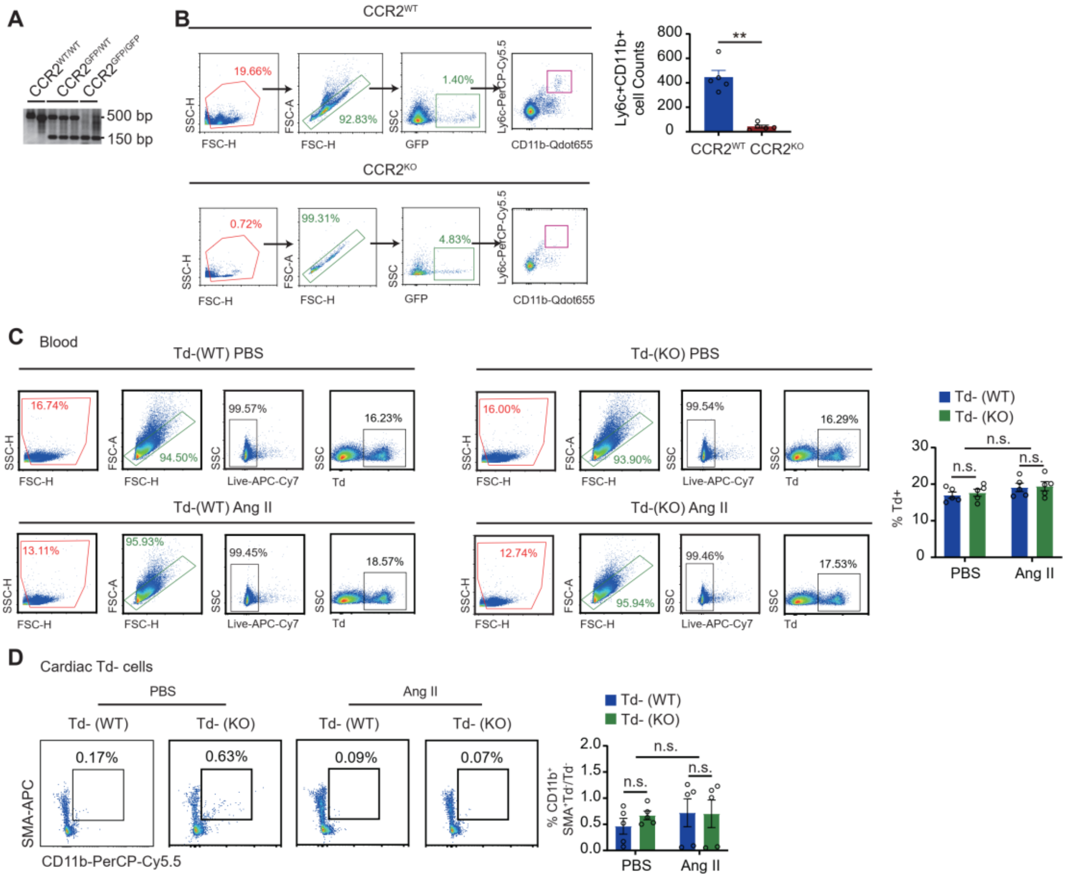

Supplementary Figure 8. CCR2 was significantly decreased in *CCR2*^{KO} mice. A, Agarose gel electrophoresis showing results of PCR genotyping. **B**, Representative flow cytometry analyses of blood CD11b+Ly6c+ cells from *CCR2*^{WT} and *CCR2*^{KO} mice with quantification at right. n=5. **C**, Representative images and quantification of flow cytometry analyses of blood Td+ cells from *CCR2*^{KO} mice cojoined with *Cx3cr1Cre; Rosa26*^{Td} or *Cx3cr1Cre; ALKBH5*$^{fl/fl}$*; Rosa26*^{Td} mice. n =5. **D**, Representative images and quantification of flow cytometry analyses of CD11b+SMA+ cells gated on Td- cells in hearts from *CCR2*^{KO} mice cojoined with *Cx3cr1Cre; Rosa26*^{Td} or *Cx3cr1Cre; ALKBH5*$^{fl/fl}$*; Rosa26*^{Td} mice. n =5. All data are presented as mean ± standard error mean. Data in (B) were analyzed by two-tailed unpaired Student's t test. Data in (C) and (D) were analyzed by two-way ANOVA followed by Tukey post-hoc tests. n.s. indicates nonsignificant. **P<0.01.

Supplementary Figure 9

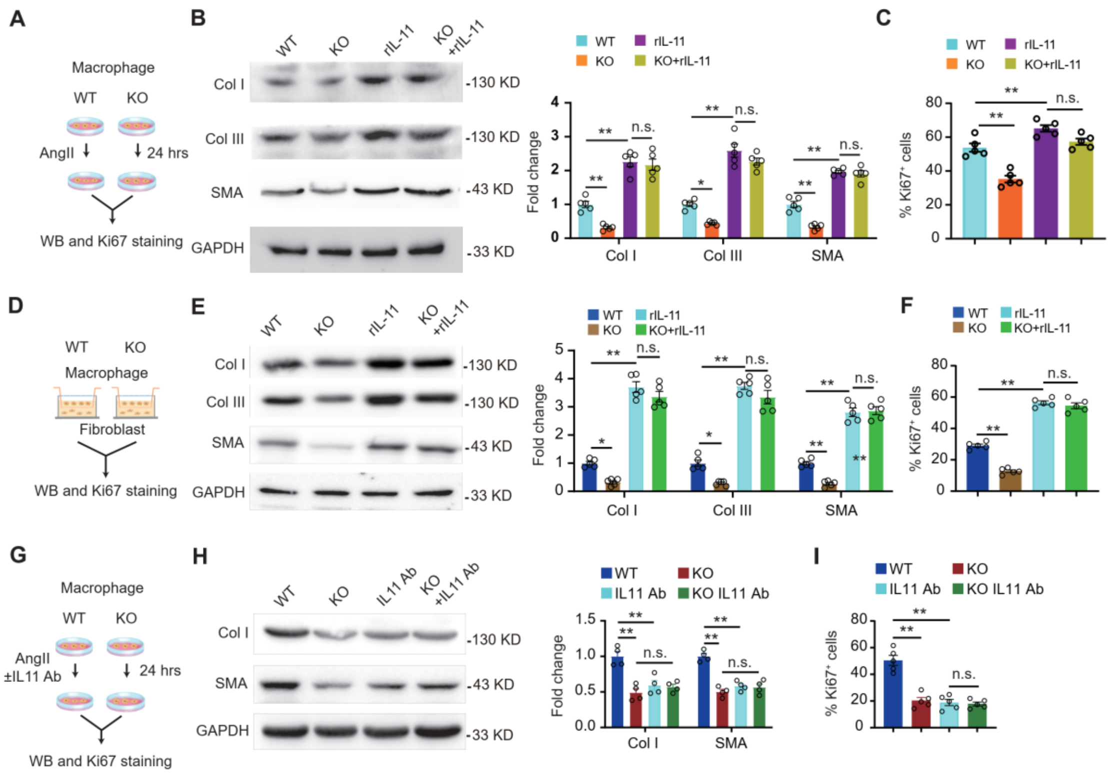

Supplementary Figure 9. Exogenous IL-11 reverses macrophage ALKBH5 deletion-mediated decrease in MMT, proliferation and ECM gene expression in myofibroblasts. A, Schematic diagram of *in vitro* experiment to show that exogenous IL-11 reversed ALKBH5 deletion-mediated decrease of MMT and macrophage proliferation with Ang II treatment. **B,** Expression of SMA and ECM genes collagen I and III in macrophages with Ang II treatment evaluated by western blot with representative images at left and quantification at right. n=5. **C,** Quantification of Ki67 positive cells in macrophages with Ang II treatment. n=5. **D,** Schematic diagram of an *in vitro* experiment to show effect of exogenous IL-11 on SMA and ECM gene expression in fibroblasts and fibroblast proliferation. **E,** Expression of SMA and ECM genes collagen I and III in fibroblasts treated with condition medium of macrophage shown by western blot with representative images at left and quantification at right (n=5). **F,** Quantification of Ki67 positive cells in fibroblasts treated with Ang II (n=5). **G**, Schematic diagram of an *in vitro* experiment to show effect of IL11 neutralizing antibody on SMA and ECM gene expression and proliferation in Ang II treated macrophages. **H** Expression of SMA and ECM gene collagen I in macrophages with Ang II treatment evaluated by western blot with representative images at left and quantification at right. n=5. **I**, Quantification of Ki67 positive cells in macrophages with Ang II treatment. n=5. All data are presented as mean ± standard error mean. Data in (B), (C), (E), (F), (H) and (I) were analyzed by one-way ANOVA followed by Tukey post-hoc tests. n.s. indicates nonsignificant. *P<0.05, **P<0.01.

Supplementary Figure 10

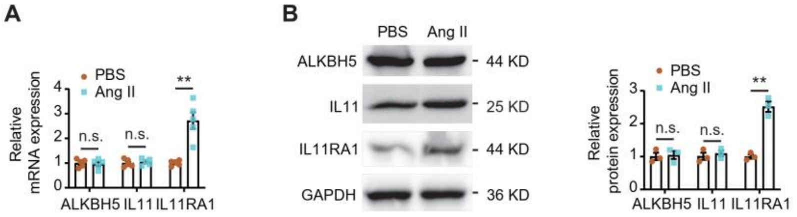

Supplementary Figure 10. ALKBH5 in fibroblasts is not changed following Ang II treatment. A, mRNA levels of ALKBH5, IL11 and IL11RA1 expression in cultured fibroblasts with and without Ang II treatment. Error bars indicate mean ± SEM. n =5. n.s. indicates nonsignificant. **P<0.01. **B**, Expression of ALKBH5, IL11 and IL11RA1 by western blot in cultured fibroblasts with and without Ang II infusion, with quantification at right. Error bars indicate mean ± SEM. n =5. **P<0.01.

Supplementary Figure 11

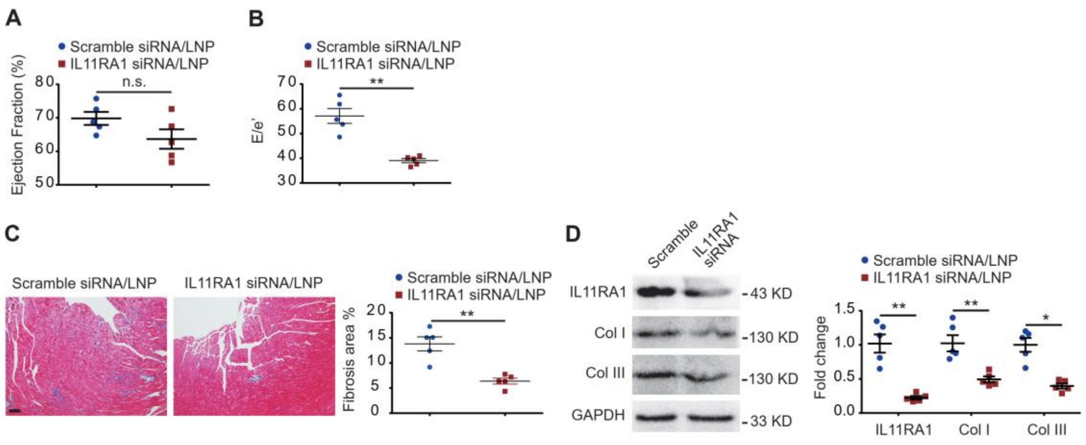

Supplementary Figure 11. Lipid nanoparticle monocyte/macrophage-target delivery of IL11RA1 siRNA attenuates Ang II-induced cardiac fibrosis and dysfunction. A, Indices of ejection fraction with scramble or IL11RA1 siRNA/LNP (n=5). Error bars indicate mean ± SEM. **B**, Indices of E/e' with scramble or IL11RA1 siRNA/LNP (n=5). Error bars indicate mean ± SEM. **C**, Representative images and quantification of positive fibrotic area of Masson trichrome staining in cardiac tissue of mice with scramble or IL11RA1 siRNA/LNP (n=5). Scale bar, 100 µm. **D**, Western blot analysis of IL11RA1, ECM genes collagen I and III in cardiac tissues from mice with scramble or IL11RA1 siRNA/LNP. Quantitative results are shown on the right (n=5). All data are presented as mean ± standard error mean. Data in (A), (B), (C) and (D) were analyzed by two-tailed unpaired Student's t test. n.s. indicates nonsignificant. *P<0.05, **P<0.01.

Supplementary Figure 12

A

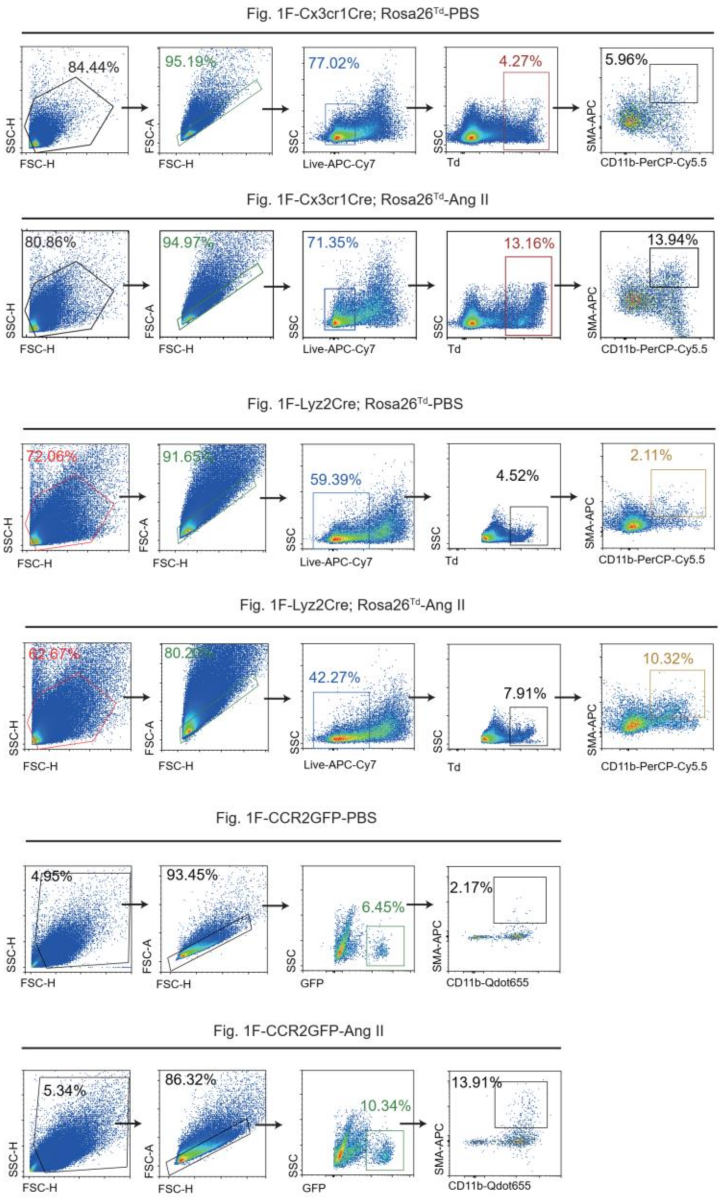

B

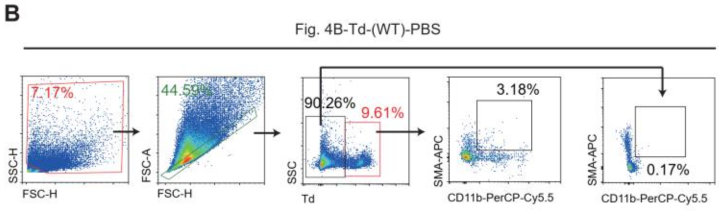

Continue

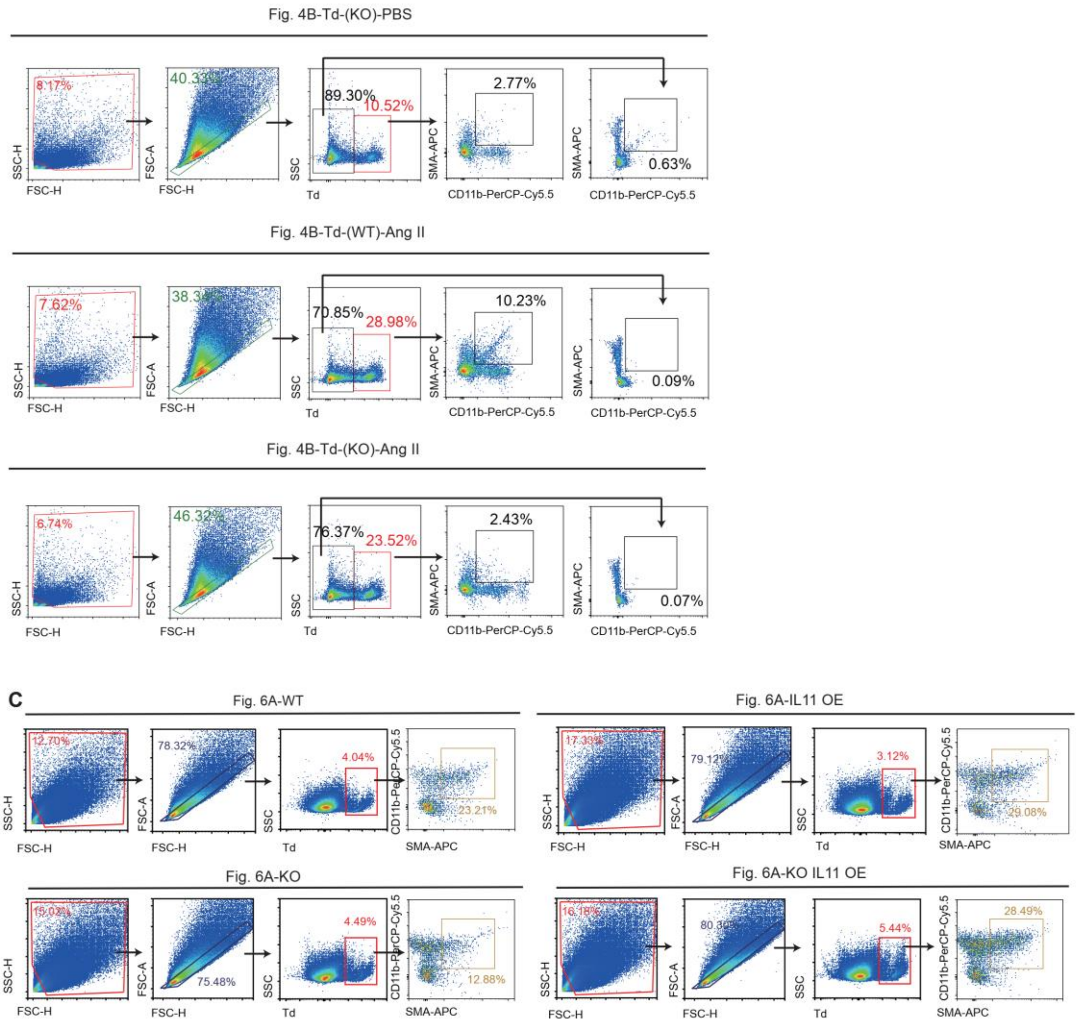

Supplementary Figure 12. Gating strategies used for flow cytometry analysis. A, Representative images of gating information for flow cytometry analysis in Figure 1F. **B**, Representative images of gating information for flow cytometry analysis in Figure 4B. **C**, Representative images of gating information for flow cytometry analysis in Figure 6A.

Supplementary Table 1: oligonucleotide sequences

Primer sequences for reverse transcript quantitative PCR (RT-qPCR)	
Gene Name	Sequence
Gapdh-F	CTA AAGGGCATCCTGGGC
Gapdh-R	TTACTCCTTGGAGGCCAT
Col I-F	CGTATCACCAAACTCAGAAG
Col I-R	TCATCATAGCCATAGGACATC
α-SMA-F	CTGAAGTATCCGATAGAACAC
α-SMA-R	AGCCTGAATAGCCACATAC
Alkbh5-F	CTTCAGATCGCCTGTCAG
Alkbh5-R	GCAATCTTCCGAGGACTC
Il-11-F	CTTCGAGTAGACTTGATGTCCTA
Il-11-R	GGGATCACAGGTTGGTCTG
Col III-F	ATGACTGTCCCACGTAAGC
Col III-R	TAGGCAATACTGTTTTTGCA
Mettl14-F	CCTATGATACATCTGCTCCAA
Mettl14-R	GTCTGTGTCCAGTGTCTAC
Mettl3-F	TAACTTACGCTGACCACTC
Mettl3-R	GTGCTTCCTTGACTTCTTAG
Wtap-F	AGTACCTCAAGCAAGTTCA
Wtap-R	TCTCCTGGATAAGCATTCG
Fto-F	CGAGACACCAGGATTAACA
Fto-R	CAACTGACAGCGTTCTAAG
Ythdc1-F	GAACCTTACAAGAGTCAACCA
Ythdc1-R	TCCTCCTCATTCTCAGTGTT
Ythdc2-F	TACTCAACCAAGACGACTAG
Ythdc2-R	GCACTTCATCCACAATAACA
Ythdf1-F	TCTCCTCTTCAGTGTCAATG
Ythdf1-R	GTTGTCGTTATTCTCCAGTC
Ythdf2-F	AATGGAGTAGGACAGTCTCA
Ythdf2-R	TCTCTTGTTACCATGCTCTG
Ythdf3-F	AGTTACGGCTATCCACCTA
Ythdf3-R	CATACCACTGCTGCTCAA
Hnrnpa2b-F	GGAGTGGAAGAGGAGGAA
Hnrnpa2b-R	AGTTAGAAGGCTGCTGGT
Primer sequences for Methylated RNA immunoprecipitation quantitative PCR	
Name	Sequence
Peak1-F	GATCGGGTTAGGAGAACAG
Peak1-R	CTCAGAGGCAGAAGAATCTA
Peak2-F	AGGTAGTCACTGCGAGAG
Peak2-R	CCTGTTGAACACGGTATGT
siRNA	
Gene Name	Sequence
ALKBH5	GCCTCAGGACATTAAGGAA
IL11RA1	CTTGGAGGAAGTGATAACA
