# Supplementary material for: ALKBH5-mediated m6A modification of IL-11 drives macrophage-to-myofibroblast transition and pathological cardiac fibrosis in mice
Source: Nat Commun. 2024 Mar 5;15:1995. doi: 10.1038/s41467-024-46357-x (PMC10914760; doi:10.1038/s41467-024-46357-x)
Supplement: Supplementary file 3 — Description of Additional Supplementary Files [file 41467_2024_46357_MOESM3_ESM.pdf]

Description of Additional Supplementary Files

Supplementary Data 1: ALKBH5 target mRNA shown by RNA immunoprecipitation-sequencing
