# Supplementary material for: ALKBH5-mediated m6A modification of IL-11 drives macrophage-to-myofibroblast transition and pathological cardiac fibrosis in mice
Source: Nat Commun. 2024 Mar 5;15:1995. doi: 10.1038/s41467-024-46357-x (PMC10914760; doi:10.1038/s41467-024-46357-x)
Supplement: Supplementary file 5 — Reporting Summary [file 41467_2024_46357_MOESM5_ESM.pdf]

nature portfolio

Corresponding author(s): Cheng-Chao Ruan

Last updated by author(s): Dec 13 2023

Reporting Summary

Nature Portfolio wishes to improve the reproducibility of the work that we publish. This form provides structure for consistency and transparency in reporting. For further information on Nature Portfolio policies, see our Editorial Policies and the Editorial Policy Checklist.

Statistics

For all statistical analyses, confirm that the following items are present in the figure legend, table legend, main text, or Methods section.

n/a	Confirmed	
☐	☒	The exact sample size (*n*) for each experimental group/condition, given as a discrete number and unit of measurement
☐	☒	A statement on whether measurements were taken from distinct samples or whether the same sample was measured repeatedly
☐	☒	The statistical test(s) used AND whether they are one- or two-sided<br>*Only common tests should be described solely by name; describe more complex techniques in the Methods section.*
☐	☒	A description of all covariates tested
☐	☒	A description of any assumptions or corrections, such as tests of normality and adjustment for multiple comparisons
☐	☒	A full description of the statistical parameters including central tendency (e.g. means) or other basic estimates (e.g. regression coefficient) AND variation (e.g. standard deviation) or associated estimates of uncertainty (e.g. confidence intervals)
☐	☒	For null hypothesis testing, the test statistic (e.g. *F*, *t*, *r*) with confidence intervals, effect sizes, degrees of freedom and *P* value noted<br>*Give P values as exact values whenever suitable.*
☒	☐	For Bayesian analysis, information on the choice of priors and Markov chain Monte Carlo settings
☒	☐	For hierarchical and complex designs, identification of the appropriate level for tests and full reporting of outcomes
☐	☒	Estimates of effect sizes (e.g. Cohen's *d*, Pearson's *r*), indicating how they were calculated

Our web collection on statistics for biologists contains articles on many of the points above.

Software and code

Policy information about availability of computer code

Data collection	sequencing data were produced by illlumina HiSeq X, NovoExpress software version 1.5.0. FlowJo 10.8 software (Becton Dickinson, United States) was used for streaming big data analysis.
Data analysis	For single-cell RNA sequencing data: Seurat package of R-project (version4.3.0).<br>MerRIP-Seq: m6A-enriched peaks in each m6AIP sample were identified using MeTDiff peak calling software version 1.1.0.<br>Differentially methylated peaks between groups were detected using MleTDif with parameter and annotated using ChIPseeker (version 12.1).<br>MEME and DREME from MEME suite (version 5.0.5) were used to detect the motif. sequence, Tomtom software from MEMEsuite (version 5.0.5) was used to annotate the motif.<br>Python (v.2.7.12) script was used for the correlation analyses of the two omics contents and to simultaneously compare transcription and methylation levels.<br>Numerical computing and statistical analysis were conducted using Graphpad 9.0<br>Western Blot was analyzed by Image J (V1.8.0). The flow cytometry results were analyzed with FlowJo 8.7 software.

For manuscripts utilizing custom algorithms or software that are central to the research but not yet described in published literature, software must be made available to editors and reviewers. We strongly encourage code deposition in a community repository (e.g. GitHub). See the Nature Portfolio guidelines for submitting code & software for further information.

Data

Policy information about availability of data

All manuscripts must include a data availability statement. This statement should provide the following information, where applicable:

- Accession codes, unique identifiers, or web links for publicly available datasets
- A description of any restrictions on data availability
- For clinical datasets or third party data, please ensure that the statement adheres to our policy

The MeRIP sequencing and single-cell RNA sequencing data as part of this study can be downloaded from Genome Sequence Archive (CRA012231). The authors declare that all other data supporting the findings of this study are available within the article and its Supplementary information files, or are avilable from the authors upon request. The source date are provided as a Source Data file.

Research involving human participants, their data, or biological material

Policy information about studies with . See also policy information about and sexual orientation and race, ethnicity and racism.

Reporting on sex and gender	N/A
Reporting on race, ethnicity, or other socially relevant groupings	N/A
Population characteristics	N/A
Recruitment	N/A
Ethics oversight	N/A

Note that full information on the approval of the study protocol must also be provided in the manuscript.

Field-specific reporting

Please select the one below that is the best fit for your research. If you are not sure, read the appropriate sections before making your selection.

☒ Life sciences ☐ Behavioural & social sciences ☐ Ecological, evolutionary & environmental sciences

For a reference copy of the document with all sections, see

Life sciences study design

All studies must disclose on these points even when the disclosure is negative.

Sample size	Sample size was chosen to ensure reproducibility of the experiments in accordance with the replacement, reduction and refinement principles of animal ethics regulation. Sample size are provided in the figure legends for each experiment and reasonable sample sizes were chosen to ensure they are sufficient for statistical comparison between different groups
Data exclusions	No data was excluded in this study.
Replication	Data are presented as means and SD of at least 3 independent experiments. All experimental findings were reliably reproduced. All experiments were performed as technical or biological replications as appropriate for the experiment design. Details of experimental replicates are given in the figure legends.
Randomization	All samples were randomly allocated into experimental groups.
Blinding	Investigators were blinded to group allocation during data collection and analysis

Reporting for specific materials, systems and methods

We require information from authors about some types of materials, experimental systems and methods used in many studies. Here, indicate whether each material, system or method listed is relevant to your study. If you are not sure if a list item applies to your research, read the appropriate section before selecting a response.

Materials & experimental systems

n/a	Involved in the study
☐	☒ Antibodies
☐	☒ Eukaryotic cell lines
☒	☐ Palaeontology and archaeology
☐	☒ Animals and other organisms
☒	☐ Clinical data
☒	☐ Dual use research of concern
☒	☐ Plants

Methods

n/a	Involved in the study
☒	☐ ChIP-seq
☐	☒ Flow cytometry
☒	☐ MRI-based neuroimaging

Antibodies

Antibodies used

For immunostaining and immunocytochemistry: anti-SMA(Cell Signaling; Cat# 19245; Clone ID: D4K9N; Host organism:rabbit; Applications: W, IP, IHC-Bond, IHC-P, IF-F.), anti-CDllb (CST; Cat# 49420; Clone ID: Clone D6X1N; Host organism:rabbit; Applications: W, IHC-Bond, IHC-P), anti-Ki67 (Abcam; Cat# ab15580; Clone ID: Clone D6X1N; Host organism:rabbit; Applications: IHC-P, ICC/IF.). 488 nm-conjugated donkey anti-rabbit secondary antibody (Invitrogen; Cat# A-21206; Clone ID: Clone D6X1N; Host organism: rabbit; Applications: ICC/IF (2 µg/mL), IHC (1-10 µg/mL), Flow (1-10 µg/mL)), 594 nm-conjugated donkey anti-rabbit secondary antibody (Invitrogen, Cat#: A21207; Clone ID: Clone D6X1N; Host organism:rabbit; Applications: Applications: Flow (1-10 µg/mL), ICC/IF (2 µg/mL), IHC (F) (1:500)), Alex FluorTM 488 Goat anti-Rabbit IgG(H+L)(Invitrogen, Cat# AllOO; Host Organism: goat; Applications: ICC/IF (4 µg/mL), Flow (1-10 µg/mL), IHC (Assay-dependent)).

For FACS: anti-live and dead (Biolegend, Cat#: 77184), anti-SMA (Proteintech; Cat# 67735-1-Ig; Clone ID: Clone 1E9A11; Host Organism: mouse; Applications: WB, IHC, IF, ELISA), anti-CDllb (Biolegend; Cat# 550993; Clone ID: M1/70; Host Organism: rat; Applications: Flow cytometry), anti-Lyvel (Invitrogen; Cat# 50-0443-82; Clone ID: Clone ALY7; Host Organism: rat; Applications: ICC/IF (1-10 µg/mL), IHC (F) (1-10 µg/mL), Flow (Assay-Dependent)), anti-Ly6c(BD pharmingenTM; Cat # 560525; Clone ID: Clone AL-21; Host Organism: rat; Applications: Flow cytometry), anti-CDllb(Biolegend; Cat#101259; Clone ID: Clone M1/70; Host Organism: rat; Applications: Flow cytometry), 647 nm-conjugated anti-mouse secondary antibody (Invitrogen; Cat# A56576; Clone ID: clone 35H35L37; Host Organism: rabbit; Applications: Flow (1-4 µg/mL), ICC/IF (0.5-4 µg/mL), WB (1-2 µg/mL)).

For Western blot: anti-Col (Abcam; Cat# ab138492; Clone ID: EPR7785; Host Organism: rabbit; Applications: WB, IHC-P), anti-Col lll (Abcam; Cat# ab184993; Clone ID: EPR17673; Host Organism: rabbit; Applications: Flow Cyt (Intra), ICC/IF, IP, WB), anti-SMA(Cell Signaling; Cat# 19245), anti-ALKBH5(Proteintech; Cat# 16837-1-AP; Host Organism: rabbit; Applications: WB, IHC, ELISA), anti-Il11RA1(Abcam; Cat# ab125015; Host Organism: rabbit;), anti-Il11(Proteintech; Cat# 55169-1-AP; Host Organism: rabbit; Applications: WB, ELISA) and anti-GAPDH(Proteintech; Cat# 10494-1-AP; Host Organism:rabbit; Applications: WB, IP, IHC, IF, FC, CoIP, ELISA), Secondary peroxidase conjugated anti-rabbit (Beyotime; Cat# A0208; Host Organism: goat; Applications: WB, ELISA, IHC) or anti-mouse (Beyotime; Cat#A0216;Applications: WB, ELISA, IHC).

Validation

All the commercially available antibodies were validated by the manufacturer via immunoblot, FACS or IF imaging

Eukaryotic cell lines

Policy information about

Cell line source(s)

The HEK 293T cell line (ATCC, CRL-1573 was purchased from ATCC.

Authentication

The cell lines were not authenticated

Mycoplasma contamination

HEK293T cell lines were tested negative for mycoplasma contamination.

Commonly misidentified lines (See ICLAC register)

No commonly misidentified cell lines are used in the study.

Animals and other research organisms

Policy information about studies involving animals; ARRIVE guidelines recommended for reporting animal research, and Sex and Gender in Research

Laboratory animals

The conditional *ALKBH5* mice (*ALKBH5*$^{flox/+}$) mice were purchased from Cyagen company (Stock#: S-CKO-09656). *Cx3cr1Cre* mice were obtained from the Jackson Laboratory (Stock#: 025524). *Lyve1Cre* mice were from the Jackson Laboratory (Stock#: 012601). Lyz2Cre (Stock#: 004781), *Cx3cr1Cre*^{ERT2} (Stock#: 021160) and *Myh11Cre*^{ERT2}(Stock#: 019079) mice were purchased from the Jackson Laboratory. *Rosa26*^{Td} (Stock#: 007914) mice were obtained from the Jackson Laboratory. *CCR2GFP* mice were from the Jackson Laboratory (Stock#: 027619). All mice were in C57BL/6J background and male mice aged 8-12 weeks were included in this study, and housed at 25℃, 12-h light/dark in a specific pathogen-free environment at the University Laboratory Animal Services Center of Fudan University. The animal experiments were approved by the Ethics Committee of Fudan University.

Wild animals

Male C57BL/6 mice purchased from SLAC Laboratory Animal Co, Ltd (Shanghai, China) were involved in this study.

Reporting on sex

The male mice were used for this study

Field-collected samples

No field collected samples were used in the study.

Ethics oversight

The animal experiments were approved by the Ethics Committee of Fudan University and were performed in accordance with the National Institutes of Health (NIH) Guide for the Care and Use of Laboratory Animals.

Note that full information on the approval of the study protocol must also be provided in the manuscript.

Flow Cytometry

Plots

Confirm that:

☒ The axis labels state the marker and fluorochrome used (e.g. CD4-FITC).

☒ The axis scales are clearly visible. Include numbers along axes only for bottom left plot of group (a 'group' is an analysis of identical markers).

☒ All plots are contour plots with outliers or pseudocolor plots.

☒ A numerical value for number of cells or percentage (with statistics) is provided.

Methodology

Sample preparation	The sample preparation was described in the Methods.
Instrument	Data were acquired on NovoCyte 3000 flow cytometer (Agilent Technologies).
Software	NovoExpress software version 1.5.0. FlowJo 10.8 software (Becton Dickinson, United States) was used for streaming big data analysis.
Cell population abundance	Hearts from Angiotensin II treated C57BL/6 mice were isolated and stored in the sCelLiveTM Tissue Preservation Solution (Singleron Bio Com, Nanjing, China) on ice within 30 mins.
Gating strategy	Cells were gated on FSC/SSC in general.

☒ Tick this box to confirm that a figure exemplifying the gating strategy is provided in the Supplementary Information.
